# A fast, simple, and cost-effective method of expanding patient-derived xenograft mouse models of pancreatic ductal adenocarcinoma

**DOI:** 10.1186/s12967-020-02414-9

**Published:** 2020-06-24

**Authors:** Zhenyang Liu, Michael Ho-Young Ahn, Tomohiro Kurokawa, Amy Ly, Gong Zhang, Fuyou Wang, Teppei Yamada, Ananthan Sadagopan, Jane Cheng, Cristina R. Ferrone, Andrew S. Liss, Kim C. Honselmann, Gregory R. Wojtkiewicz, Soldano Ferrone, Xinhui Wang

**Affiliations:** 1 Division of Surgical Oncology, Department of Surgery, Massachusetts General Hospital, Harvard Medical School, Boston, MA USA; 2 Department of Gastroenterology and Urology and of Medical Oncology, Hunan Cancer Hospital, Xiangya School of Medicine, Central South University, Changsha, Hunan China; 3 Department of Pathology, Massachusetts General Hospital, Harvard Medical School, Boston, MA USA; 4 Division of General and Gastrointestinal Surgery, Department of Surgery, Massachusetts General Hospital, Harvard Medical School, Boston, MA USA; 5 Mouse Imaging Program, Center for Systems Biology, Massachusetts General Hospital, Harvard Medical School, Boston, MA USA; 6 Department of Orthopaedic Surgery, Massachusetts General Hospital, Harvard Medical School, Boston, MA USA

**Keywords:** PDX expansion, Subcutaneous PDX mouse model, Orthotopic PDX mouse model, PDAC PDX

## Abstract

**Background:**

Patient-derived xenograft (PDX) mouse models of cancer have been recognized as better mouse models that recapitulate the characteristics of original malignancies including preserved tumor heterogeneity, lineage hierarchy, and tumor microenvironment. However, common challenges of PDX models are the significant time required for tumor expansion, reduced tumor take rates, and higher costs. Here, we describe a fast, simple, and cost-effective method of expanding PDX of pancreatic ductal adenocarcinoma (PDAC) in mice.

**Methods:**

We used two established frozen PDAC PDX tissues (derived from two different patients) and implanted them subcutaneously into SCID mice. After tissues reached 10–20 mm in diameter, we performed survival surgery on each mouse to harvest 90–95% of subcutaneous PDX (incomplete resection), allowing the remaining 5–10% of PDX to continue growing in the same mouse.

**Results:**

We expanded three consecutive passages (P1, P2, and P3) of PDX in the same mouse. Comparing the times required for in vivo expansion, P2 and P3 (expanded through incomplete resection) grew 26-60% faster than P1. Moreover, such expanded PDX tissues were successfully implanted orthotopically into mouse pancreases. Within 20 weeks using only 14 mice, we generated sufficient PDX tissue for future implantation of 200 mice. Our histology study confirmed that the morphologies of cancer cells and stromal structures were similar across all three passages of subcutaneous PDX and the orthotopic PDX and were reflective of the original patient tumors.

**Conclusions:**

Taking advantage of incomplete resection of tumors associated with high local recurrence, we established a fast method of PDAC PDX expansion in mice.

## Background

Pancreatic cancer is one of the most common and deadly types of cancer. In the United States, pancreatic cancer is currently the fourth leading cause of cancer-related death for both men and women [1] and is projected to become the second leading cause of cancer-related death by 2030 [2]. Pancreatic cancer has an extremely poor prognosis with a 5-year survival rate of 9%, the lowest of all major cancers [3]. Pancreatic ductal adenocarcinoma (PDAC) accounts for 95% of all pancreatic cancers [4]. Surgery is the only treatment that could possibly cure pancreatic cancer, but less than 10% of all pancreatic cancer patients have tumors that are potentially curable with resection, while more than 50% have disease that is considered locally advanced, unresectable pancreatic cancer (LAPC) [5]. Other treatments for pancreatic cancer are limited to radiation therapy and chemotherapy. However, the role of radiation therapy in treating LAPC remains controversial [6] and pancreatic cancer can develop resistance to chemotherapy, thereby limiting its therapeutic effects [7–9]. Thus, the mechanisms of formation, development, progression, and novel treatment of PDAC merit further investigation.

Mouse models can be powerful tools for preclinical studies of cancer. However, results from preclinical studies do not always correlate with those from human clinical trials. One study that simulated combination trials with the human-specific anti-VEGF antibody bevacizumab found that half of the genetically engineered mouse (GEM) models of PDAC showed improved survival [10], whereas the corresponding phase III trial of the Cancer and Leukemia Group B (CALGB 80303) concluded that the addition of bevacizumab to gemcitabine does not improve survival in advanced pancreatic cancer patients [11]. Late-stage human clinical trial failures like this urgently call for improved mouse models that better reflect human disease, particularly diseases with poor prognoses such as pancreatic cancer.

The three main types of preclinical mouse cancer models include the cell line-derived xenograft (CDX) model, the genetically engineered mouse (GEM) model, and the patient-derived xenograft (PDX) model. All three models have their individual merits and limitations, but PDX models arguably have the highest predictive value and human relevance [12]. The discrepancy between promising preclinical studies and unsuccessful clinical trials may lie in preclinical mouse models that do not accurately reflect human disease [13]. CDX models may not be representative of the original tumor in its native state, especially if the cell lines used for the CDX have been passaged for several generations [14]. GEM models are very useful for studying the role of specific genes in tumor development and progression, but they cannot capture the complexity of the human tumor and can take as long as a year to create [15]. Although inherently limited by their immunodeficiency, PDX models translate well in clinical trials [16] since they maintain the molecular, genetic, and histological heterogeneity of the original human tumor through serial passages in mice [17]. Among PDX models, orthotopic PDX models have been shown to better mimic metastasis than subcutaneous PDX models [18] because subcutaneously implanted PDX tissue rarely metastasizes [19]. Therefore, research results derived from orthotopic PDX models have high translational value.

The current trend in cancer research is to develop and use orthotopic PDX mouse models, as several studies have already established orthotopic PDX mouse models for various cancers including breast cancer [20], cervical cancer [21], lung cancer [22], ovarian cancer [23], pancreatic cancer [24, 25], soft tissue sarcoma [26], and renal cell carcinoma [27]. However, there are still many questions that need to be addressed, such as how to set up standard procedures for PDX establishment and whether authentic histological characteristics can be maintained in orthotopic implantations and subcutaneous implantations through serial passages in mice.

The major limitations of using orthotopic PDX models are cost and time. Although PDX models of PDAC are commercially available, they can be quite costly. Some companies may not even permit researchers to passage and expand the PDX tissue in mice independently. Other companies may require researchers to outsource the study to the company, thus limiting researchers’ autonomy and control over the study. Since it can be very expensive and time-consuming to carry out experiments involving PDX models, we aimed to develop a fast, simple, and cost-effective method of expanding PDX in mice and establishing an orthotopic PDX mouse model of PDAC.

## Methods

### PDX tissues

Two PDX tissues were obtained from the Liss Laboratory, which operates the Pancreatic Tumor Bank at the Massachusetts General Hospital. This clinically annotated biobank contains both normal tissue and tumor tissue, as well as white blood cells, serum, and plasma, from more than 2,600 patients [28]. Both PDX tissues are from non-treated patients with PDAC and measured about 6-7 mm in diameter. No genetic information is available for either tissue. Patient 1275 PDX tissue was derived from a liver metastasis of a 70-year-old man with a primary PDAC lesion in the pancreatic tail. It weighed 0.399 g and was the 12th passage in nude mice from the original patient tumor. Patient 1319 PDX tissue was derived from a poorly differentiated T3N1M0 tumor of the head of the pancreas of a 66-year-old man with PDAC. It weighed 0.251 g and was the 11th passage in nude mice from the original patient tumor.

### Mice

Male severe combined immunodeficient (SCID) mice, 6-8 weeks of age, were obtained from the Cox 7 Gnotobiotic Animal Facility at the Massachusetts General Hospital. All animal experiments were performed with permission from the Institutional Animal Care and Use Committee (IACUC) at the Massachusetts General Hospital.

### Reagents

- IsoFlo^®^ Isoflurane, USP (Abbott Laboratories, Cat. No. 5260-04-05)
- Ketofen^®^ Ketoprofen Injection (Zoetis Inc., Cat. No. NDC 54771-4396-1)
- Phosphate Buffered Saline, 0.01 M, pH 7.4 (Sigma-Aldrich Corporation, Cat. No. P3813)
- Cellgro™ DMEM, 1X with 4.5 g/L glucose, l-glutamine & sodium pyruvate (Mediatech Inc., Cat. No. 10-013-CV)
- Cellgro™ RPMI 1640, 1X with l-glutamine (Mediatech Inc., Cat. No. 10-040-CV)
- Penicillin–Streptomycin Solution, 100X (Corning Inc., Cat. No. 30-002-Cl)
- Bacteriostatic 0.9% Sodium Chloride Injection, USP (Pfizer Inc., Cat. No. 00409-1966-07)
- BenchMark™ Fetal Bovine Serum (Gemini Bio-Products, Cat. No. 100-106)
- Dimethyl Sulfoxide (Fisher Scientific Inc., Cat. No. BP231-1)
- Freezing Medium (90% Benchmark™ Fetal Bovine Serum + 10% Dimethyl Sulfoxide)
- Matrigel^®^ Matrix, Basement Membrane High Concentration (HC), Phenol-Red Free, LDEV Free (Corning Inc., Cat. No. 354262)
- PROTOCOL™ 10% Buffered Formalin, Fisher Diagnostics™ (Fisher Scientific Inc., Cat. No. 23-245685)
- Povidone-Iodine Prep Pads (Dynarex Corporation, Cat. No. 1108)
- Ethanol 200 Proof, Anhydrous (Decon Laboratories Inc.)
- Isopropyl Alcohol (Sigma-Aldrich Corporation)
- Milli-Q^®^ Type 1 Ultrapure Water System (Merck Group)
- Ice
- Dry Ice
- Magnevist^®^ Gadopentetate Dimeglumine (Bayer AG)
- Paraffin (Sigma-Aldrich Corporation)
- Dako Hematoxylin (Agilent Technologies Inc.)
- Eosin (Sigma-Aldrich Corporation)
- Richard-Allan Scientific™ Cytoseal™ 60 (Thermo Fisher Scientific Inc.)

### Equipment

- Delicate Operating Scissors, Straight, Sharp–Sharp, 30 mm Blade Length, 4 ¾” Overall Length (Roboz Surgical Instrument Co., Cat. No. RS-6702)
- Operating Scissors, Straight, Sharp–Sharp, 4.5” Length (Roboz Surgical Instrument Co., Cat. No. RS-6802)
- Thumb Dressing Forceps, Serrated, 4.5” Length, 2.2 mm Tip Width (Roboz Surgical Instrument Co., Cat. No. RS-8100)
- Graefe Forceps, Straight, Serrated (Fine Science Tools Inc., Cat. No. 11050-10)
- Graefe Forceps, Curved, Serrated (Fine Science Tools Inc., Cat. No. 11051-10)
- Iris Forceps, Straight, Serrated (Fine Science Tools Inc., Cat. No. 11064-07)
- Iris Forceps, Curved, Serrated (Fine Science Tools Inc., Cat. No. 11065-07)
- Codman^®^ Crile Classic^®^ Delicate Hemostatic Forceps, 5 ½” straight (Johnson & Johnson Inc., Cat. No. 30-4467)
- Round Handled Needle Holders (Fine Science Tools Inc., Cat. No. 12075-12)
- Chex-All^®^ II Self-Seal Sterilization Pouches (Propper Manufacturing Co. Inc., Cat. No. 02400800)
- Sterile Cotton Tipped Applicator (Puritan Medical Products Co., Cat. No. 25-826 5WC)
- MiniArco™ Rechargeable Trimmer (Wahl Clipper Corporation, Cat. No. 8787-450A)
- 4-0 MONOCRYL^®^ Undyed Monofilament 27” PS-2 Reverse Cutting, Sterile, Poliglecaprone 25 Absorbable Surgical Suture (Ethicon Inc., Cat. No. Y426H)
- 8-0 PROLENE^®^ Blue Monofilament 5” BV130-5 Taper, Sterile, Polypropylene Nonabsorbable Surgical Suture (Ethicon Inc., Cat. No. 2775G)
- Falcon^®^ Bacteriological Petri Dishes, 100 mm x 15 mm, Not TC-Treated Polystyrene, Sterile (Corning Inc., Cat. No. 351029)
- Nalgene^®^ System 100™ Cryogenic Tubes, 1.5 mL (Thermo Fisher Scientific Inc., Cat. No. 5000-1020)
- Nalgene^®^ Mr. Frosty Cryo 1 °C Freezing Container (Thermo Fisher Scientific Inc., Cat. No. 5100-0001)
- Bel-Art^®^ SP Scienceware™ Magic Touch™ Ice Bucket, 2.5 Liter (SP Industries Inc., Cat. No. M18848-2002)
- Syringe with Luer-Lok™ Tip, 30 mL, Sterile (BD, Cat. No. 302832)
- PrecisionGlide™ Needle, 18G x 1, 1.2 mm x 25 mm, Sterile (BD, Cat. No. 305195)
- Portable Pipet-Aid^®^ XP Pipette Controller (Drummond Scientific Co., Cat. No. 4-000-101)
- Falcon^®^ 2 mL Serological Pipet, Polystyrene, 0.01 Increments, Individually Packed, Sterile, 100/Box, 1000/Case (Corning Inc., Cat. No. 357507)
- Falcon^®^ 10 mL Serological Pipet, Polystyrene, 0.1 Increments, Individually Packed, Sterile, 50/Bag, 200/Case (Corning Inc., Cat. No. 357551)
- Falcon^®^ 25 mL Serological Pipet, Polystyrene, Space Saver, 0.25 Increments, Sterile, 50/Pack, 200/Case (Corning Inc., Cat. No. 357525)
- Falcon^®^ 50 mL High Clarity PP Centrifuge Tube, Conical Bottom, Sterile, 25/Rack, 500/Case (Corning Inc., Cat. No. 352098)
- Small Animal Heated Pad, 9” x 12” (K&H Manufacturing LLC, Cat. No. 1060)
- Denver Instrument™ Balance Model XL-3K (Denver Instrument Co., Cat. No. XL-3K)
- Fisherbrand™ Polystyrene Antistatic Weighing Dishes (Fisher Scientific Inc., Cat. No. 08-732-112)
- V-1 Tabletop Laboratory Animal Anesthesia System (VetEquip Inc., Cat. No. 901806)
- Medical USP Grade Oxygen, Size E Aluminum Cylinder, CGA-870 (Airgas Inc., Cat. No. OX USPEA)
- Oxygen Regulator, E Cylinder (VetEquip Inc., Cat. No. 901305)
- Oxygen Cylinder Wrench with Security Chain (VetEquip Inc., Cat. No. 201744)
- Economy Oxygen E or D Cylinder Cart (WT Farley Inc., Cat. No. CR-HC300)
- LabGard^®^ ES NU-602 Class II, Type A2 Animal Handling Biological Safety Cabinet (NuAire Inc., Cat. No. NU-602)
- LabGard^®^ NU-425-300 Class II, Type A/B3 Biological Safety Cabinet (NuAire Inc., Cat. No. NU-425-300)
- Allegra^®^ 6R Benchtop Centrifuge, Refrigerated, 60 Hz, 120 V (Beckman Coulter Inc., Cat. No. 366816)
- Isotemp™ Digital-Control Water Bath: Model 205 (Fisher Scientific Inc., Cat. No. 15-462-5Q)
- 4.7 Tesla MRI System (Bruker Corporation)
- OsiriX Software (Pixmeo SARL)
- Microm^®^ HM 325 Rotary Microtome (Thermo Fisher Scientific Inc., Cat. No. 902100)
- Olympus™ BX51 Microscope (Olympus Corporation)

### Subcutaneous PDX tissue implantation

1. Autoclave all surgical instruments for sterilization
2. If the PDX tissue is fresh, then:
  - Use a syringe to wash away the blood from the PDX tissue with 1X PBS containing 1X penicillin–streptomycin (PS-PBS)
  - Measure the weight of the PDX tissue
  - For short-term storage, use forceps to place the PDX tissue in a 50 mL tube on ice containing 20 mL of DMEM or RPMI 1640
  - Use forceps to place the PDX tissue on a petri dish on ice containing PS-PBS
  - Use a scalpel to cut the PDX tissue into 2-3 mm diameter pieces
3. If the PDX tissue is frozen, then:
  - Retrieve a cryogenic tube of frozen PDX tissue from the liquid nitrogen (LN_2_) freezer and temporarily store on dry ice
  - Quickly thaw the cryogenic tube in a 37 °C water bath
  - Use forceps to transfer the PDX tissue to a 50 mL tube
  - Use a Pipet-Aid^®^ to wash away the freezing medium from the PDX tissue with 20 mL of PS-PBS
  - Centrifuge at 500x*g* (4 °C) for 3 minutes
  - Discard the supernatant
  - Repeat Steps 3d–3f
  - Measure the weight of the PDX tissue
  - Use forceps to place the PDX tissue on a petri dish on ice containing PS-PBS
  - Use a scalpel to cut the PDX tissue into 2–3 mm diameter pieces
4. Prepare another petri dish on ice that contains 1 mL of Matrigel^®^ Matrix (Matrigel^®^ Matrix must be placed on ice for thawing and at all times to prevent solidification)
5. Use isoflurane to anesthetize one mouse, 6–8 weeks of age, and place the anesthetized mouse in the prone position on a heated pad in the Animal Handling Biological Safety Cabinet
6. Use an electric trimmer to trim the fur on the right lower quadrant of the dorsal side of the mouse, then disinfect the skin with povidone-iodine and 70% ethanol
7. Use scissors to make a skin incision (≤ 5 mm) on the right lower flank
8. Use forceps to lift the skin and use scissors to create a pocket in the subcutaneous space
9. Use forceps to submerge one piece of PDX tissue in Matrigel^®^ Matrix, then subcutaneously implant the PDX tissue into the created pocket
10. Suture the skin incision with 4-0 MONOCRYL^®^ absorbable suture
11. Use forceps to transfer 4–5 pieces of remaining PDX tissue per cryogenic tube containing 600–700µL of freezing medium
12. Freeze the cryogenic tubes with a Nalgene^®^ freezing container (filled with isopropyl alcohol) in −80 °C for at least 24 hours, then store the cryogenic tubes in the LN_2_ freezer for future PDX tissue implantations
13. Repeat Steps 1–12 for additional subcutaneous PDX tissue implantations

### Subcutaneous PDX tissue harvest

14. Autoclave all surgical instruments for sterilization
15. After about 7–8 weeks when the subcutaneous PDX tissue has grown to 10-20 mm in diameter, perform Steps 5–8 to prepare one mouse for subcutaneous PDX tissue harvest
16. Use scissors and forceps to harvest 90–95% of the subcutaneous PDX tissue (incomplete resection), then place it on a petri dish on ice containing PS-PBS
17. Perform Step 10 to suture the skin incision
18. Perform Step 2, but cut the subcutaneous PDX tissue into 1–1.5 mm diameter pieces (See Step 24 to begin performing the Orthotopic PDX Tissue Implantation using this first passage (P1) of harvested subcutaneous PDX tissue)
19. Perform Steps 11–12 to freeze and store any remaining pieces of subcutaneous PDX tissue
20. Perform Steps 14–19 for additional subcutaneous PDX tissue harvests

### Orthotopic PDX tissue implantation

21. Autoclave all surgical instruments for sterilization
22. If the PDX tissue is fresh, then perform Step 2, but cut the PDX tissue into 1–1.5 mm diameter pieces
23. If the PDX tissue is frozen, then perform Step 3, but cut the PDX tissue into 1–1.5 mm diameter pieces
24. Prepare another petri dish on ice that contains 1 mL of Bacteriostatic 0.9% Sodium Chloride Injection
25. Use isoflurane to anesthetize one mouse, 6–8 weeks of age, and place the anesthetized mouse in the supine position on a heated pad in the Animal Handling Biological Safety Cabinet
26. Use an electric trimmer to trim the fur on the left upper quadrant of the ventral side of the mouse, then disinfect the skin with povidone-iodine and 70% ethanol
27. Use scissors to make a skin incision (≤ 5 mm) on the left upper quadrant of the abdomen
28. Use another pair of sterile scissors to make a peritoneal incision underneath the skin incision
29. Use forceps and a cotton tipped applicator to gently externalize the spleen and expose the pancreas
30. Use forceps to create a small pocket in the tail of the pancreas, then place one piece of subcutaneous PDX tissue in the created pocket
31. Suture the created pocket with 8-0 PROLENE^®^ nonabsorbable suture (nonabsorbable sutures do not release anti-inflammatory factors that may damage the pancreas)
32. Use a cotton tipped applicator to scrub the peritoneal incision with Bacteriostatic 0.9% Sodium Chloride Injection (Bacteriostatic 0.9% Sodium Chloride Injection helps prevent the PDX tissue from attaching to the peritoneum)
33. Perform Step 10 to suture the skin incision
34. Perform Steps 11–12 to freeze and store any remaining pieces of PDX tissue
35. Perform Steps 21–34 for additional orthotopic PDX tissue implantations

### Orthotopic PDX tissue harvest

36. Autoclave all surgical instruments for sterilization
37. After about 8–12 weeks when the orthotopic PDX tissue has grown to 10–20 mm in diameter, perform Steps 25–28 to prepare one mouse for orthotopic PDX tissue harvest
38. Use scissors and forceps to harvest all of the orthotopic PDX tissue, then place it on a petri dish on ice containing PS-PBS
39. Euthanize the mouse with CO_2_
40. Perform Step 2, but cut the orthotopic PDX tissue into 1–1.5 mm diameter pieces
41. Perform Steps 11–12 to freeze and store the pieces of orthotopic PDX tissue
42. Perform Steps 36–41 for additional orthotopic PDX tissue harvests

### Postoperative recovery

Unconscious mice were not left unattended. All mice were kept warm using heated pads intraoperatively and postoperatively. All postoperative mice regained consciousness within 15–60 minutes.

### Postoperative analgesic treatment

Analgesics were provided for all mice following postoperative survival. To this end, all mice were given ketoprofen, 5 mg/kg subcutaneously once to twice daily for 3 days.

### Magnetic resonance imaging (MRI)

Mice were anesthetized using ~ 2% isoflurane in oxygen and imaged on a Bruker 4.7 Tesla MRI with a T1 rapid acquisition with relaxation enhancement (RARE) sequence (TE: 13.59 ms, TR: 900 ms, Avg: 8, RARE Factor: 4, Matrix: 256 x 256 x 18, Voxel Size: 0.156 mm x 0.156 mm x 1.0 mm) and a T2 RARE sequence (TE: 60 ms, TR: 4500 ms, Avg: 6, RARE Factor: 10, Matrix: 256 x 256 x 18, Voxel Size: 0.156 mm x 0.156 mm x 1.0 mm) before injection of 15 µl of Magnevist^®^ gadopentetate dimeglumine contrast agent with an additional T1 RARE sequence immediately post-injection. Images were created in OsiriX software.

### Hematoxylin and Eosin (H&E) staining

The PDX tissues were fixed in 10% buffered formalin for 24 hours then paraffin embedded by the Histopathology Research Core at the Massachusetts General Hospital. The tissue blocks were cut into 4–5 µm sections using a rotary microtome then mounted on slides and deparaffinized. After a graded series of rehydration, sections were stained with hematoxylin and eosin then coverslipped with Cytoseal™ 60.

## Results

### Three consecutive passages of subcutaneous PDX tissue successfully expanded in the same mouse

**Fig. 1:**
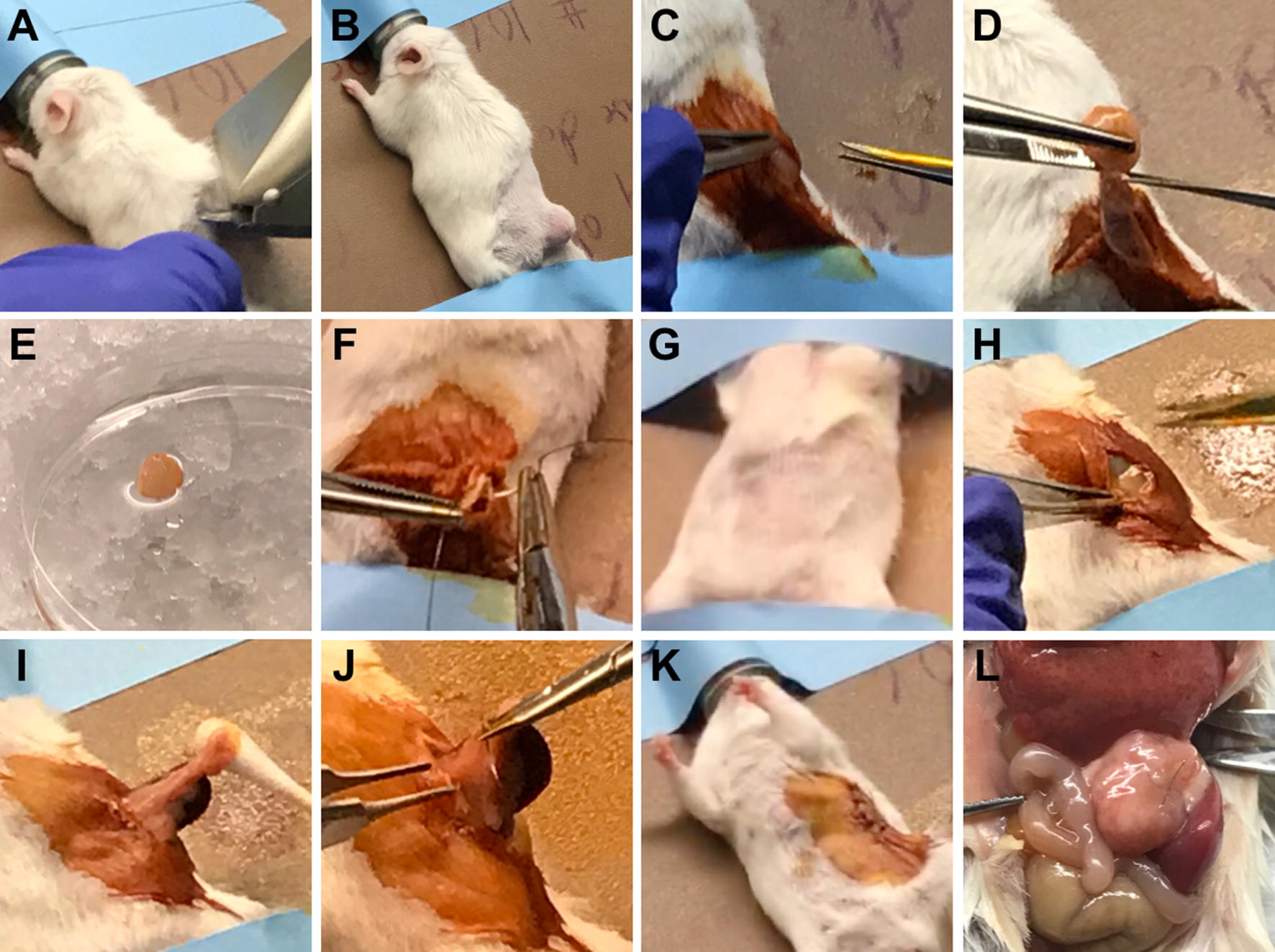
Surgical Procedures for Subcutaneous PDX Tissue Harvest and Orthotopic PDX Tissue Implantation. **a** Trimming the right lower quadrant of the dorsal side of the anesthetized mouse (prone position). **b** Subcutaneous PDX tissue exposed after trimming the fur **c** Using scissors to make a skin incision (≤ 5 mm) and separate the subcutaneous tissue from the disinfected skin on the right lower flank. **d** Using scissors and forceps to harvest 90-95% of the subcutaneous PDX tissue (incomplete resection). **e** Harvested subcutaneous PDX tissue placed on a petri dish on ice containing PS-PBS. **f** Suturing the skin incision with 4-0 MONOCRYL^®^ absorbable suture. **g** Trimming the left upper quadrant of the ventral side of the anesthetized mouse (supine position). **h** Using scissors to make a skin incision (≤ 5 mm) on the left upper quadrant of the abdomen. **i** Using a cotton tipped applicator to gently externalize the spleen and expose the pancreas. **j** Suturing the created pocket in the tail of the pancreas (containing one piece of P1 subcutaneous PDX tissue) with 8-0 PROLENE^®^ nonabsorbable suture. **k** Suturing the skin incision with 4-0 MONOCRYL^®^ absorbable suture. **l** PDX tissue successfully expanded orthotopically in the pancreas

A total of six male SCID mice received subcutaneous PDX tissue implantations, of which three mice received Patient 1275 PDX tissue implantations, while another three mice received Patient 1319 PDX tissue implantations. Three consecutive passages (P1, P2, and P3) of subcutaneous PDX tissue were successfully expanded in the same mouse for all six mice (Fig. 1).

### Successful orthotopic implantation using subcutaneously expanded PDX tissue

**Fig. 2:**
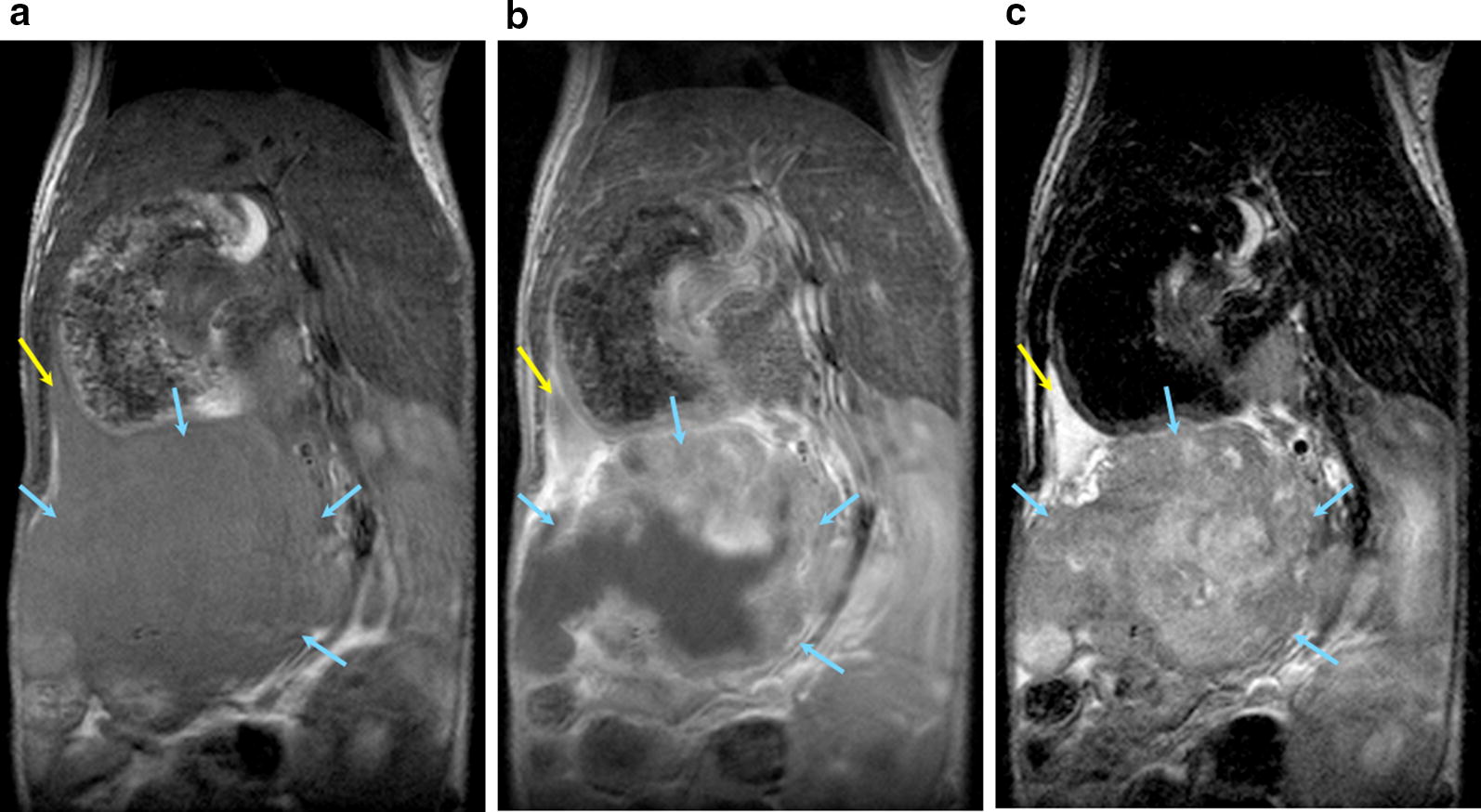
Detection of Orthotopic Pancreatic PDX Tumor by MRI. Yellow: Pancreas; Blue: Tumor. T1-weighted pre-contrast (**a**), post-contrast (**b**), and T2-weighted (**c**) magnetic resonance images of coronal cross sections of the mouse demonstrate successful orthotopic implantation of Patient 1275 PDX tissue into the mouse pancreas. The high signal intensity of the tumor in the T1-weighted post-contrast image indicates high levels of vascularization. The high intensity regions in the T2-weighted image of the pancreas and the tumor suggest the increased presence of fluid

A total of eight male SCID mice received orthotopic PDX tissue implantations using the first passage (P1) of subcutaneously expanded PDX tissue, of which four mice received Patient 1275 PDX tissue implantations, while another four mice received Patient 1319 PDX tissue implantations (Fig. 1). MRI scans were performed for one live mouse that received an orthotopic Patient 1275 PDX tissue implantation. The MRI study confirmed that the Patient 1275 PDX tissue was successfully implanted and expanded orthotopically in the mouse pancreas (Fig. 2).

### Patient 1319 PDX tissue expanded faster than Patient 1275 PDX tissue

**Fig. 3:**
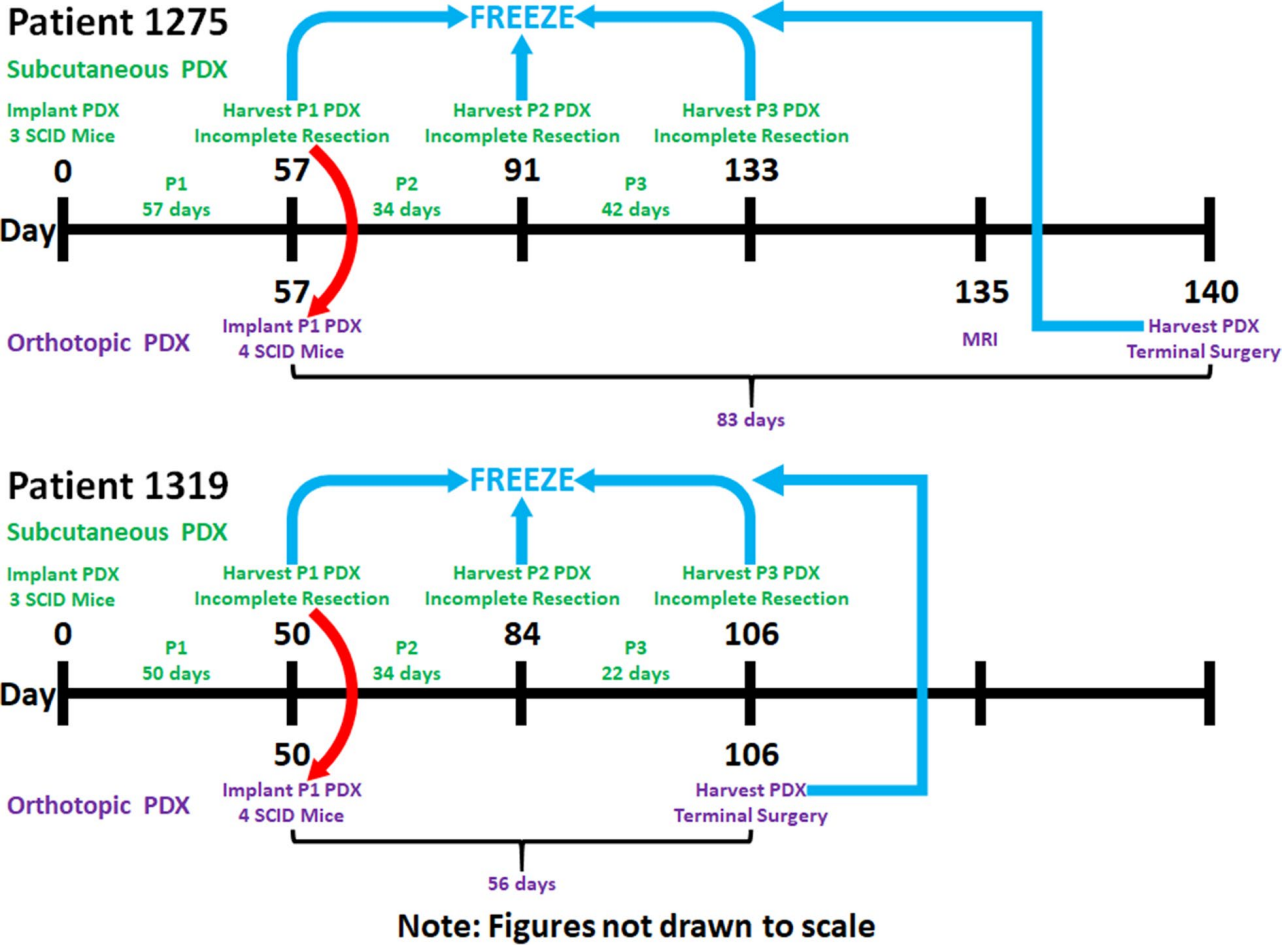
Timeline of PDX Tissue Implantations and Harvests. PDX: patient-derived xenograft; P1: first passage; P2: second passage; P3: third passage; MRI: magnetic resonance imaging. Green: subcutaneous PDX tissue procedures; Purple: orthotopic PDX tissue procedures; Red: using P1 subcutaneous PDX tissue for orthotopic implantation; Blue: freezing (−80 °C) and storing (LN_2_) PDX tissues

After subcutaneously implanting Patient 1275 PDX tissue in three male SCID mice, it took 57 days (about 8 weeks) to expand the first passage (P1) of subcutaneous PDX tissue, then 34 days (about 5 weeks) to expand the second passage (P2) of subcutaneous PDX tissue, then 42 days (6 weeks) to expand the third passage (P3) of subcutaneous PDX tissue. All subcutaneous PDX tissues were expanded to a diameter of 10-20 mm. After orthotopically implanting Patient 1275 PDX tissue in four male SCID mice, it took 83 days (about 12 weeks) to expand the orthotopic PDX tissue to a diameter of 20 mm (Fig. 3).

After subcutaneously implanting Patient 1319 PDX tissue in three male SCID mice, it took 50 days (about 7 weeks) to expand the first passage (P1) of subcutaneous PDX tissue, then 34 days (about 5 weeks) to expand the second passage (P2) of subcutaneous PDX tissue, then 22 days (about 3 weeks) to expand the third passage (P3) of subcutaneous PDX tissue. All subcutaneous PDX tissues were expanded to a diameter of 10–20 mm. After orthotopically implanting Patient 1319 PDX tissue in four male SCID mice, it took 56 days (8 weeks) to expand the orthotopic PDX tissue to a diameter of 20 mm (Fig. 3).

Overall, it took 133 days (19 weeks) to expand three consecutive passages of subcutaneous PDX tissue with Patient 1275 PDX tissue (Table 1), while it took 106 days (about 15 weeks) to expand three consecutive passages of subcutaneous PDX tissue with Patient 1319 PDX tissue (Table 2). Comparing the times required for in vivo expansion, P2 and P3 (expanded through incomplete resection) grew 26–60% faster than P1 (Table 3).

**Table 1:** Timeline of Patient 1275 PDX Tissue Implantations and Harvests

Day	Subcutaneous PDX Tissue	Orthotopic PDX Tissue
0	Subcutaneous PDX tissue implantation	
57	Incomplete resection to harvest first passage (P1) of subcutaneous PDX tissue	Orthotopic PDX tissue implantation using P1 subcutaneous PDX tissue
91	Incomplete resection to harvest second passage (P2) of subcutaneous PDX tissue	
133	Incomplete resection to harvest third passage (P3) of subcutaneous PDX tissue	
135		MRI
140		Terminal surgery to harvest orthotopic PDX tissue

PDX: patient-derived xenograft; P1: first passage; P2: second passage; P3: third passage; MRI: magnetic resonance imaging

**Table 2:** Timeline of Patient 1319 PDX Tissue Implantations and Harvests

Day	Subcutaneous PDX Tissue	Orthotopic PDX Tissue
0	Subcutaneous PDX tissue implantation	
50	Incomplete resection to harvest first passage (P1) of subcutaneous PDX tissue	Orthotopic PDX tissue implantation using P1 subcutaneous PDX tissue
84	Incomplete resection to harvest second passage (P2) of subcutaneous PDX tissue	
106	Terminal surgery to harvest third passage (P3) of subcutaneous PDX tissue	Terminal surgery to harvest orthotopic PDX tissue

PDX: patient-derived xenograft; P1: first passage; P2: second passage; P3: third passage

**Table 3:** Less Time Required for in vivo Expansion of P2 and P3 Subcutaneous PDX Tissues

Passage	Patient 1275	Patient 1319
Days Required for in vivo Expansion of Subcutaneous PDX Tissues		
P1	57	50
P2	34	34
P3	42	22

PDX: patient-derived xenograft; P1: first passage; P2: second passage; P3: third passage

### Subcutaneously and orthotopically expanded PDX tissues sufficient for future implantation of 200 mice

Within 20 weeks using a total of 14 male SCID mice, two PDX tissues were subcutaneously and orthotopically expanded to generate sufficient PDX tissue for future implantation of 200 mice.

### Histological features of PDX tissues remain the same across passages and location of implantation

**Fig. 4:**
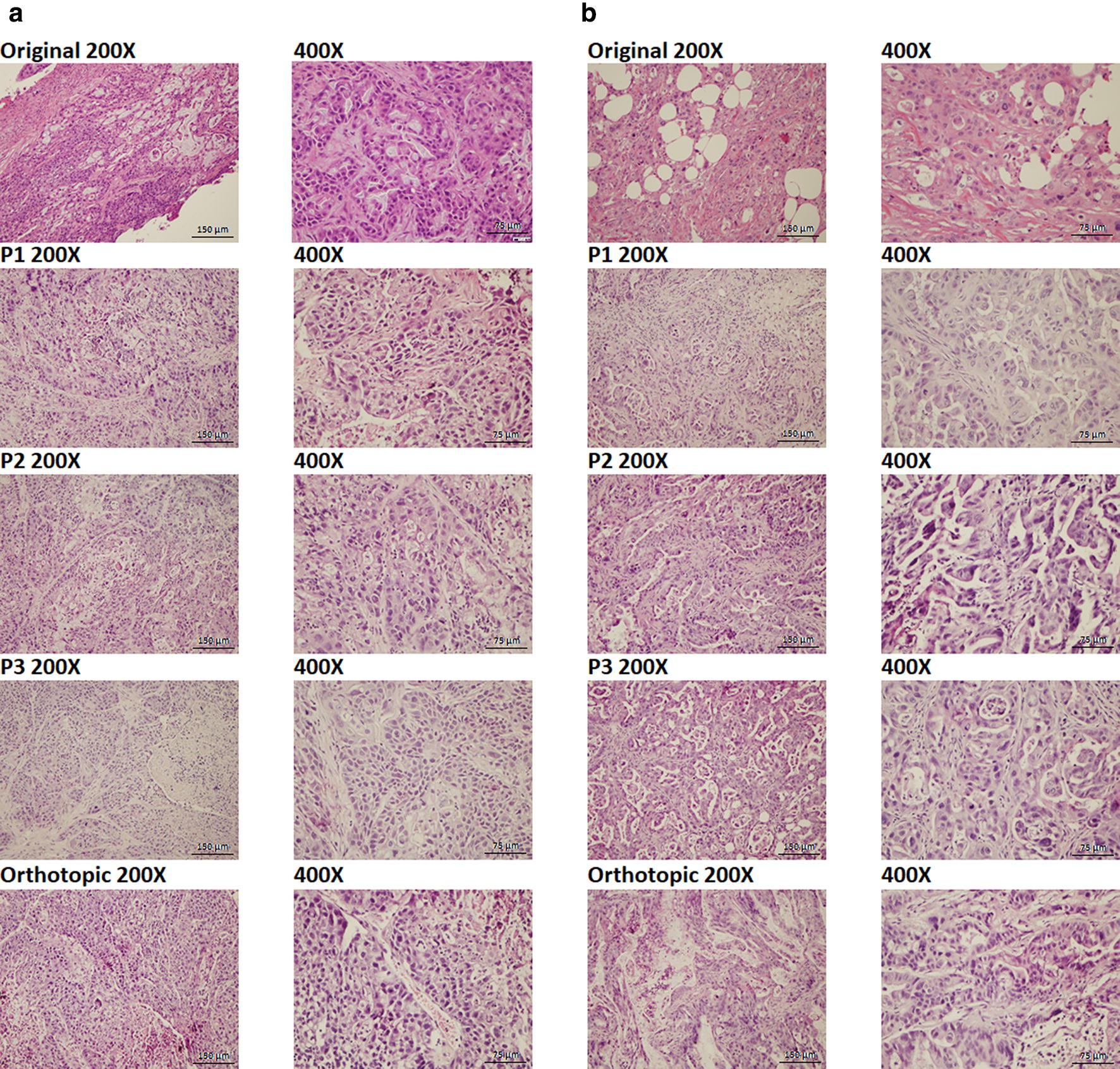
Similar Histological Features Shared by All PDX Tissues and the Original Patient PDAC Tumors. The original poorly differentiated PDAC tumors from patients were histologically compared with their derived Patient 1275 PDX Tissue (**a**) or Patient 1319 PDX Tissue (**b**). Both PDX tissues were respectively passaged and expanded subcutaneously three times in the same mouse through incomplete resection, then engrafted and expanded orthotopically one time in the pancreatic tail of another mouse. Pictures were taken at magnifications indicated using an Olympus™ BX51 microscope

Histological evaluation demonstrated morphologic similarity among all three passages of subcutaneous PDX tissue, the orthotopic PDX tissue, and the original patient PDAC tumors (Fig. 4).

## Discussion

Methods of establishing orthotopic PDX models of PDAC have been previously described [29]. A recent study revealed that orthotopic PDX models of pancreatic cancer parallel human disease by exhibiting metastasis and inducing muscle wasting that resembles cancer cachexia syndrome [30], supporting the idea that orthotopic PDX models are preferable to subcutaneous PDX models for studying metastasis of primary tumors [18, 19]. Studies have also shown that the molecular profiles of both subcutaneous and orthotopic PDX models of PDAC remain stable after extensive passages [31] and that the orthotopic PDX model of PDAC closely recapitulates the clinical, pathologic, genetic, and molecular aspects of human disease [32]. Another recent study showed that biomarker expression was significantly higher in orthotopic PDX models compared to subcutaneous PDX models, stressing the importance of the orthotopic tumor microenvironment when evaluating the clinical relevance of novel biomarkers [33]. The benefits of using orthotopic PDX models of PDAC are great, but these models remain very expensive, time-consuming, and labor-intensive to create [34]. Our study aimed to address the financial and logistical challenges of carrying out experiments involving orthotopic PDX models.

Within 20 weeks using a total of 14 male SCID mice, we expanded two PDX tissues to generate sufficient PDX tissue for an estimated 200 future mouse implantations. Since the PDX tissues originated from male patients, we used male mice to match the biological sex. It took about 7–8 weeks to expand the first passage of subcutaneous PDX tissue followed by about 3–6 weeks per subsequent passage of subcutaneous PDX tissue and about 8–12 weeks to expand the orthotopic PDX tissue. The reason we were able to quickly generate an abundance of PDX tissue in a relatively short amount of time is that all three passages of subcutaneous PDX tissue were expanded in the same mice that originally received the subcutaneous PDX tissue implantations. By performing survival surgery and incomplete resection to allow the remaining 5–10% of subcutaneous PDX tissue to continue growing in the same mouse, it was easy to continuously expand the subcutaneous PDX tissue for three consecutive passages. Storing the harvested PDX tissues in liquid nitrogen allows for long-term storage and gives researchers freedom and convenience to plan and conduct experiments on their own schedule.

The MRI study confirmed that the PDX tissue of PDAC was successfully implanted and expanded orthotopically in the mouse pancreas. The histology study demonstrated that the morphologies of all PDX tissues were similar to those of the original PDX tissue and remained the same throughout all three passages of subcutaneous PDX tissue and the orthotopic PDX tissue. Since histological features of the PDX tissue remain the same across passages, this model can be widely used for preclinical studies such as predicting response to therapy [35] and tracing tumor growth and metastasis [36]. However, further studies must be performed to assess whether the transcriptome and the genetic drift of the PDX tissues are also maintained through all passages. Moreover, histological and genetic features should be compared between passages of PDX tissue expanded using the method presented by this study and those expanded (from the same original PDX tissue and equally passaged through mice) using the current traditional method of PDX expansion. The latter would serve as a control in drawing a convincing conclusion as to how comparable the passages of PDX tissue expanded by both methods are.

The importance of our study is that it provides a method of producing PDX models in a cost- and time-efficient manner. While PDX models are commercially available, they are often quite expensive. But performing in vivo experiments requires several mice (e.g., ≥ 5 mice per group) to obtain statistically significant data. In addition, orthotopic PDX models are currently not as readily available on the market compared to subcutaneous PDX models. Our study offers researchers flexibility and independence by allowing them to create and expand their own PDX models in a timely manner at a relatively low cost.

The main limitation of our study is that we used PDX tissues originating from only two patients. Although these two PDX tissues had highly successful engraftment rates and reasonably fast expansion times, they grew at different rates: Patient 1319 PDX tissue grew faster than Patient 1275 PDX tissue. This variation in growth rate can be due to uncontrollable factors such as the original tumor aggressiveness and size in the patient [37]. In addition, it would be remiss not to acknowledge the inherent limitation of PDX models for immunotherapy research as the mice bearing PDX are immunodeficient.

## Conclusions

Through incomplete resection of PDX tumors in mice, we established a fast, simple, and cost-effective method of expanding PDX tissue of PDAC in mice. Our method offers the advantage of using fewer mice and taking less time in expanding PDX tissues for all related research purposes, not only for PDAC but perhaps for other types of cancer as well.

## Data Availability

The datasets used and analyzed during the current study are available from the corresponding author upon reasonable request.

## Abbreviations

CDX: Cell line-derived xenograft
GEM: Genetically engineered mouse
LAPC: Locally advanced, unresectable pancreatic cancer
LN_2_: Liquid nitrogen
MRI: Magnetic resonance imaging
P1: First passage
P2: Second passage
P3: Third passage
PDAC: Pancreatic ductal adenocarcinoma
PDX: Patient-derived xenograft
PS-PBS: 1X phosphate-buffered saline containing 1X penicillin–streptomycin
RARE: Rapid acquisition with relaxation enhancement
SCID: Severe combined immunodeficient
